# The Impact of Documentation Training on Performance Reporting

**DOI:** 10.7759/cureus.283

**Published:** 2015-07-10

**Authors:** Terrence Loftus, Hadi Najafian, Sushil R Pandey, Paravasthu Ramanujam

**Affiliations:** 1 Care Management, Banner Health; 2 West Valley Colon & Rectal Surgery Center, Banner Boswell Medical Center

**Keywords:** clinical coding, data quality, data reporting, documentation, quality indicators, quality improvement, clinical documentation, performance improvement

## Abstract

With the advent of public reporting of clinical performance for physicians, the need for accurate documentation is essential. This study tested the hypothesis that a short tutorial on five key documentation tips for a group of colorectal surgeons could significantly improve their reported clinical performance. Data was collected on a total of 626 consecutive inpatients before and after the introduction of a short tutorial focusing on five key documentation tips to a group of colorectal surgeons. Quality metrics were compared between the two time periods. Significant improvements were observed for complications (p = 0.001), morbidity (p = 0.046), ileus (p = 0.027), and digestive system complications (p < 0.01). There was no difference in mortality (p = 0.569) or readmissions (p = 0.920). A short tutorial focusing on five key documentation tips is associated with improvement in the reported clinical performance of colorectal surgeons.

## Introduction

In 2010, the Affordable Care Act (ACA) required The Centers for Medicare and Medicaid Services (CMS) to create a Physician Compare website to be used for public reporting of clinical performance[1]. Their plan was to begin public reporting of quality data for physicians during the calendar year of 2014. This data is derived from CMS’s claims data which is based on hospital’s administrative data. The administrative data is based on coding which is a reflection of the accuracy of the documentation in a patient’s chart. Discrepancies in the documentation could therefore lead to inaccurate performance reporting of physicians.

Just as medical knowledge has advanced and become more complex over the years, coding of this knowledge has also become more complex. The transition from ICD-9 to ICD-10 alone will increase procedure and diagnosis codes from 17,849 to 141,747 [2]. Recently, the validity of reports derived from administrative data has been questioned due to inaccurate and inconsistent coding as reflected by the documentation [3]. Zalatimo, et al. [4] concluded that a simple educational intervention could improve the accuracy of documentation and quality metrics for a group of neurosurgeons. In this study, we tested the hypothesis that a short tutorial on five key documentation tips for a group of colorectal surgeons could significantly improve their reported clinical performance.

## Materials and methods

This was a retrospective study comparing the before and after results of common quality metrics following a brief educational intervention focusing on five key documentation tips with three colorectal surgeons in a large community hospital. Data was collected on a total of 626 consecutive inpatients during the time period of 11 months prior to and 10 months after the educational intervention. Patients included were adults, 18 years of age and older, admitted to one hospital by this same group. The rates for morbidity, mortality, readmissions, and complications, along with four specific complications: anemia, digestive system complications, ileus, and hypokalemia, were compared between the two time periods. These four complications were chosen since they were noted as the most frequent complications identified for this group of colorectal surgeons.

The educational intervention consisted of a one-hour tutorial covering five key documentation tips. The tutorial was presented by Clinical Documentation Specialists who are nurses trained in coding and routinely work with physicians around coding and documentation clarifications. The five key tips taught in the tutorial are listed in Table 1 and adapted from the official guidelines [5].

**Table 1:** Five Key Documentation Tips

Documentation Tip	Example
If it is documented, then it will be coded.	Coders can use any diagnosis to identify complications associated with the operation. If the patient’s potassium is low by laboratory standards but it is not clinically significant, then physicians will oftentimes document this for completeness, which is not necessary since it does not impact care.
Do not document a condition unless it is clinically significant, requires additional treatment and/or prolongs LOS.	Many patients following an abdominal procedure will have a “physiological ileus” which is an expected part of the procedure and does not require documentation. If the ileus persists and leads to a longer length of stay, then it is a “pathological ileus” and should be documented.
If it is clinically expected, state it.	If a patient undergoes a Hartmann’s procedure for perforated diverticulitis and is being admitted to the Intensive Care Unit for management of respiratory failure “due to” septic shock “due to” perforated diverticulitis, then state this in the operative report or progress note. Don’t assume the coder will make this clinical connection. If they don’t, then the respiratory failure may get counted as a complication of your operation.
If it is present on admission, state it.	If a patient undergoes a colectomy for colon cancer and was anemic prior to the operation, then be sure to associate the post-operative anemia as being “due to” the present on admission diagnosis of anemia “due to” colon cancer (i.e. anemia in neoplastic disease). This assumes there wasn’t a clinically significant event (acute post hemorrhagic anemia) to explain the post-operative anemia.
Be aware of consultant’s notes.	Consultants can be helpful in the management of patients, however remember to review their notes and make sure they are not documenting events that are not clinically significant.

This study was determined to be exempt by Banner Health's Institutional Review Board (Project # 01-13-0108, Reference # 013699). Statistical analyses were performed using the Z-Test for two population proportions. A p value < 0.05 was considered statistically significant.

## Results

The outcomes of 300 patients (baseline period) were compared to 326 patients (study period). Following introduction of a one-hour tutorial on five key documentation tips, there were significant improvements observed for complications (p = 0.001), morbidity (p = 0.046), ileus (p = 0.027), and digestive system complications (p < 0.01). There was a trend in improvement for anemia (p = 0.087) and hypokalemia (p = 0.060). There was no difference in mortality (p = 0.569) or readmissions (p = 0.920) (Table 2).

**Table 2:** Results

Condition	Baseline (%)	10 month (%)	Z-score	p-value
Anemia	11.50%	7.50%	1.7118	0.087
Complications	63.90%	50.30%	3.4318	0.001
Dig system comp	12.50%	0.00%	6.5838	< 0.01
Hypokalemia	13.40%	8.70%	1.8811	0.06
Morbidity	21.27%	15.11%	2.0014	0.046
Mortality	1.00%	0.60%	0.5673	0.569
Paralytic ileus	14.70%	9.00%	2.214	0.027
Readmissions	15.30%	15.00%	0.1046	0.92

## Discussion

The public reporting of physician quality performance is a key component of the ACA. Surgeons are concerned regarding the validity of the metrics being used [6]. This study demonstrates that these concerns may be valid. A one-hour tutorial focusing on five key documentation tips resulted in a significant improvement in complications and morbidity. This suggests that it is not just a physician’s clinical practice that influences the quality metrics being used for public reporting; there is also a documentation practice that matters.

Medical knowledge continues to evolve, as does the necessary documentation and coding rules that accompany these changes. Physicians are often required to complete continuing medical education (CME) to maintain licensure and board certification; however, there is no requirement for physicians to keep up with the most recent rule changes for coding. While the American College of Surgeons (ACS) sponsors coding workshops and updates [7], participation is voluntary.  Subtle aspects of coding rules can potentially introduce noise into the quality reporting system. For example, if a surgeon documents the presence of a postoperative ileus (physiologic) but does not distinguish this from a pathologic ileus, then it will coded as a complication and not considered a routine finding after a major abdominal operation. While such nuances may seem absurd to surgeons, coding rules allow for such determinations unless the surgeon who performs the operation clarifies this in their documentation.

While the number of codes will increase with the introduction of ICD-10, physicians can take relief in recognizing that memorizing all these codes is not necessary. As this study suggests, it is probably more important to understand the specific rules that impact one's practice. We targeted rules that impacted the reported complication rate for colorectal surgeons and found a significant reduction or trend for all complication types except mortality and readmissions. Not too surprising, documentation will not affect whether a patient dies or is readmitted. One of the limitations of this study is whether these rates would have been maintained when risk-adjusted. While there was no specific change made in the group’s clinical practice or patient selection, there is the risk that both of these elements could be confounding variables. If not, then the difference between the baseline and study results represents the noise of documentation that exists in public reporting. This noise will persist until better methods for tracking clinical outcomes, such as the ACS’s National Surgical Quality Improvement Program’s (NSQIP) database [8], become utilized for such purposes. In the short-term, surgeons are best advised to collaborate with their coding specialists in order to provide the best documentation that accurately reflects the condition and care of their patients.

## Conclusions

A short tutorial focusing on five key documentation tips reduces the noise of documentation inaccuracies and improves the reported clinical performance for surgeons. From a public reporting perspective, it may become essential for surgeons to include continuing coding education along with their usual CME activities.

## References

[1] (2014). Physician Compare Overview. http://www.cms.gov/Medicare/Quality-Initiatives-Patient-Assessment-Instruments/physician-compare-initiative/Physician-Compare-Overview.html.

[2] (2014). International Classification of Diseases, (ICD-10-CM/PCS) Transition. http://www.cdc.gov/nchs/icd/icd10cm_pcs_background.htm.

[3] Knightly JJ, Meyer SA, Weiss BB, Bustami R, Halperin JJ, Fox S, Diamond M (2013). 117 accurately dead or alive: A neurosurgical review of quality patient care and outcomes. The importance of data fidelity in calculating quality metrics utilizing University Health System Consortium (UHC) Clinical Administrative Database. Neurosurg.

[4] Zalatimo O, Ranasinghe M, Harbaugh RE, Iantosca M (2014). Impact of improved documentation on an academic neurosurgical practice. J Neurosurg.

[5] (2014). ICD-9-CM Official Guidelines for Coding and Reporting. http://www.cdc.gov/nchs/data/icd/icd9cm_guidelines_2011.pdf.

[6] Sherman KL, Gordon EJ, Mahvi DM, Chung J, Bentrem DJ, Holl JL, Bilimoria KY (2013). Surgeons' perceptions of public reporting of hospital and individual surgeon quality. Med Care.

[7] Savarise MT (2013). Coding for hospital admission, consultations, and emergency department visits. Bull Am Coll Surg.

[8] (2014). ACS NSQIP. http://site.acsnsqip.org/.

